# The role of reactive oxygen species in the pathogenesis and treatment of endometrial cancer

**DOI:** 10.3389/fmed.2025.1662794

**Published:** 2025-10-10

**Authors:** Jeongmin Lee, Seung Geun Yeo, Jae Min Lee, Sung Soo Kim, You Jeong Jeong, Tong In Oh, Dong Choon Park

**Affiliations:** ^1^Department of Medicine, College of Medicine, Kyung Hee University Medical Center, Seoul, Republic of Korea; ^2^Department of Otorhinolaryngology Head & Neck Surgery, College of Medicine, Kyung Hee University Medical Center, Seoul, Republic of Korea; ^3^Department of Precision Medicine, Graduate School, Kyung Hee University, Seoul, Republic of Korea; ^4^Department of Convergence Medicine, College of Medicine, Kyung Hee University, Seoul, Republic of Korea; ^5^Medical Research Center for Bioreaction to Reactive Oxygen Species and Biomedical Science Institute, Core Research Institute, Kyung Hee University, Seoul, Republic of Korea; ^6^Department of Biochemistry and Molecular Biology, College of Medicine, Kyung Hee University, Seoul, Republic of Korea; ^7^Department of Biomedical Engineering, College of Medicine, Kyung Hee University, Seoul, Republic of Korea; ^8^Department of Obstetrics and Gynecology, St. Vincent’s Hospital, College of Medicine, The Catholic University of Korea, Seoul, Republic of Korea

**Keywords:** reactive oxygen species (ROS), endometrial cancer, oxidative stress, therapeutic targets, hormonal regulation

## Abstract

Reactive oxygen species (ROS) play dual roles in the pathophysiology of endometrial cancer (EC). Oxidative stress induced by ROS can promote the survival and proliferation of cancer cells, whereas excessive accumulation of ROS can lead to various forms of programmed cell death—including ferroptosis and apoptosis—making ROS potential therapeutic targets in cancer treatment. EC is the most common gynecologic malignancy in developed countries, and its global incidence and mortality rates have been steadily increasing. Although significant research has been conducted on the etiology and treatment of EC, progress remains limited. Thus, further exploration of the role of ROS in the pathogenesis of EC is warranted. In this study, we conducted a literature review using databases including the Cochrane Library, EMBASE, Google Scholar, PubMed, and SCOPUS with the search terms “endometrial cancer” and “nitric oxide.” Of the 142 identified articles, 18 were selected for detailed review. The analysis revealed that ROS contributes to EC progression through mechanisms such as DNA damage and genomic instability, interactions with estrogen and progesterone signaling, and immune dysregulation. Potential therapeutic agents targeting ROS identified in the literature include hinokitiol, *α*-terthienylmethanol, ellipticine, fructose-1,6-bisphosphate, oleanolic acid 3-acetate, CaBP-28 k, simvastatin, and amentoflavone. These findings suggest that oxidative stress plays a critical role in the progression of EC. A deeper understanding of ROS regulatory mechanisms may open new avenues for the development of targeted therapies for EC.

## 1 Introduction

### 1.1 Endometrial cancer

Endometrial cancer (EC) is the most common gynecologic malignancy in developed countries, with its incidence and mortality rates increasing globally. This trend is particularly pronounced in countries undergoing rapid socioeconomic transitions. Traditionally, EC has been classified into two subtypes—Type I and Type II—based primarily on histopathological features (1). Type I EC accounts for approximately 80% of all cases and is typically estrogen-dependent. It is commonly associated with mutations in PTEN (phosphatase and tensin homolog), PIK3CA (phosphatidylinositol-4,5-bisphosphate 3-kinase catalytic subunit alpha), and the proto-oncogene KRAS, and generally carries a more favorable prognosis. In contrast, Type II EC is estrogen-independent, frequently harbors TP53 mutations, exhibits high chromosomal instability, and is considered more aggressive (2).

More recently, the Cancer Genome Atlas (TCGA) project proposed a molecular classification system that stratifies EC into four distinct subgroups: POLE-ultramutated, microsatellite instability-high (MSI-H or MMRd), copy-number low (NSMP), and copy-number high (p53-abnormal) (Cancer Genome Atlas Research Network, 2013). This molecular taxonomy has significantly improved prognostic accuracy and guided more tailored therapeutic approaches. The ProMisE (Proactive Molecular Risk Classifiers for Endometrial Cancer) and TransPORTEC (Translational research in Post Operative Radiation Therapy in Endometrial Carcinoma) studies have further validated the TCGA classification and supported its integration into updated clinical treatment guidelines (3, 4). The development of EC is closely linked to hormonal, genetic, and metabolic factors. Major risk factors include obesity, hyperinsulinemia, chronic inflammation, prolonged estrogen exposure (e.g., in polycystic ovary syndrome or tamoxifen use), and hereditary conditions such as Lynch syndrome (5–7). Among these, obesity is considered one of the most significant contributors. In adipose tissue, androgens are converted to estrogens, leading to sustained estrogen exposure, which in turn promotes endometrial hyperplasia and increases cancer risk. Additionally, hyperinsulinemia and insulin resistance are known to activate the phosphoinositide 3-kinase (PI3K)/AKT/mTOR signaling pathway, thereby facilitating the growth and progression of EC (8, 9).

Recent studies have revealed that oxidative stress and reactive oxygen species (ROS) play critical roles in the initiation and progression of EC. ROS induce genomic instability through DNA damage, lipid peroxidation, and protein modifications, thereby activating oncogenic signaling pathways. Moreover, ROS contribute to tumor metastasis and resistance to chemotherapy by regulating inflammatory cytokines, angiogenic factors (e.g., VEGF), and epithelial–mesenchymal transition (EMT) inducers (10, 11). Diagnostic tools for EC include histopathological evaluation, imaging modalities such as magnetic resonance imaging (MRI) and ultrasound, and molecular biomarker profiling. Recently, immunohistochemical targeting of markers like TP53, POLE (DNA polymerase epsilon), and MMR (mismatch repair) proteins, as well as next-generation sequencing (NGS), have become essential for guiding therapeutic decisions (2). Standard treatment for early-stage EC involves total hysterectomy with bilateral salpingo-oophorectomy; adjuvant radiotherapy or chemotherapy may be indicated in high-risk cases (9). For advanced or recurrent disease, emerging therapies showing promising results include immune checkpoint inhibitors (PD-1/PD-L1 inhibitors such as pembrolizumab and dostarlimab) and HER2-targeted agents (e.g., trastuzumab) (4, 6).

### 1.2 Reactive oxygen species

Reactive oxygen species (ROS) are a group of chemically highly reactive molecules formed as a consequence of the electron-accepting properties of oxygen. They include hydrogen peroxide (H_2_O_2_), superoxide anion (O2^−^), hydroxyl radical (∙OH), and singlet oxygen. ROS are produced as by-products of normal aerobic metabolic processes that are essential for energy production and metabolic regulation, primarily in cellular organelles such as mitochondria, peroxisomes, and chloroplasts, with mitochondria being the major source. In particular, superoxide anions can be generated during ATP production in the mitochondrial electron transport chain (ETC). Under normal conditions, oxygen is reduced to water, but a portion of the oxygen is incompletely reduced, leading to the formation of ROS. Complexes I and III of the ETC are known to leak electrons to oxygen, producing superoxide. In addition, ROS are continuously produced by oxidative enzymes such as NADPH oxidase (NOX), xanthine oxidase, cytochrome P450, and enzymes in peroxisomes and the endoplasmic reticulum (ER).

ROS play crucial roles in cellular signaling, contributing to physiological processes such as cell proliferation, differentiation, and immune regulation. They can modulate gene expression through activation of transcription factors such as NF-κB (nuclear factor kappa-light-chain-enhancer of activated B cells), AP-1, and HIF-1α (hypoxia-inducible factor 1 alpha), and influence intracellular calcium signaling, protein phosphorylation, and growth factor pathways. Recent studies suggest that the actions of ROS as signaling molecules depend on their concentration, localization, and persistence (12, 13).

However, when ROS levels are pathologically elevated or antioxidant defense systems are impaired, ROS can cause extensive damage to key cellular components including DNA, proteins, and lipids—a condition referred to as oxidative stress (14) that leads to cellular dysfunction and the onset of various diseases (15, 16). Such damage includes DNA double-strand breaks, base oxidation, protein carbonylation, and lipid peroxidation, which disrupt cellular structure and function and can ultimately lead to apoptosis or altered survival pathways associated with malignancy. In particular, ROS contribute to carcinogenesis by modulating multiple signaling pathways such as NF-κB, MAPK (mitogen-activated protein kinase), PI3K/AKT, and p53, thereby promoting inflammation, increasing genomic instability, and shaping a tumor-promoting microenvironment (17–19).

ROS generation in the tumor microenvironment is significantly increased due to a combination of factors, including high metabolic activity, mitochondrial dysfunction, infiltration of inflammatory cells, and hypoxia. In particular, ROS production by tumor-associated immune cells is closely linked to immunosuppression. For instance, excessive ROS can suppress T cell activity and enhance the function of tumor-derived myeloid-derived suppressor cells (MDSCs), thereby facilitating immune evasion (20, 21). These characteristics suggest that ROS play a dual role in various tumor pathologies, including EC: on one hand, ROS can be induced by chemotherapy or radiotherapy to promote cancer cell death; on the other, they may contribute to cancer cell survival, metastasis, and treatment resistance.

As a result, therapeutic strategies targeting ROS are generally divided into two categories. The first is antioxidant therapy aimed at suppressing or eliminating ROS production. The second involves increasing ROS generation beyond the oxidative stress threshold of cancer cells, thereby inducing cytotoxicity. In fact, several anticancer drugs are based on ROS induction; more recently, precisely controlled platforms such as ROS-scavenging biomaterials have also been developed (22–24).

## 2 Methods

**Figure 1:**
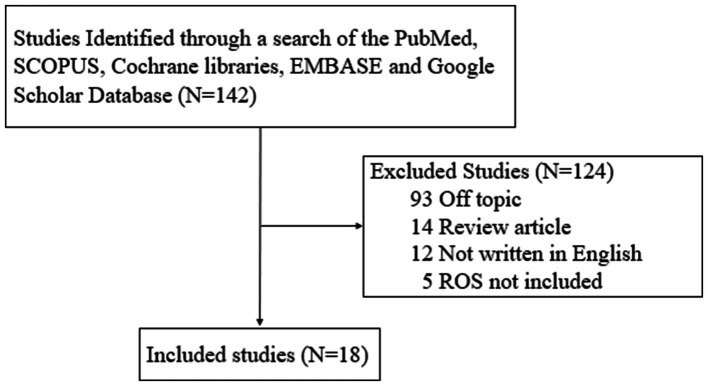
Review flow diagram.

Although various studies have examined the role of ROS in different diseases, the role of ROS in the development of EC has not been the subject of an extensive review. To address this gap, we performed a comprehensive review of the literature on the subject. One author (J.L.) searched for studies published between January 1990 and March 2025 in five electronic databases—Cochrane Library, EMBASE, Google Scholar, PubMed, and SCOPUS—using the search terms “endometrial cancer” and “nitric oxide.” The literature search focused on studies published in English and included: (1) prospective or retrospective studies on ROS in endometriosis, and (2) studies involving either humans or animals. The following were excluded: (1) unpublished data, (2) review articles, (3) gray literature, (4) case reports, and (5) duplicates. A total of 142 articles were initially retrieved, 18 of which satisfied the screening criteria and were ultimately included in this literature review (Figure 1).

## 3 Discussion

Endometrial cancer (EC) is a common gynecologic malignancy, and accumulating evidence implicates reactive oxygen species (ROS) as key regulators of its initiation and progression. ROS are generated during normal cellular metabolism and, at physiological levels, contribute to signaling, immune defense, and gene regulation. However, when ROS exceed homeostatic thresholds, they provoke oxidative stress that can drive DNA damage, alter signaling networks, and promote tumorigenic processes. In this review, we synthesize current data on how ROS shape EC pathophysiology—spanning interactions with hormonal signaling, immune modulation, and stress pathways—and evaluate therapeutic approaches that either exploit ROS-induced cytotoxicity or mitigate pathogenic oxidative stress. In this study, we sought to investigate the role of ROS in the pathophysiology and progression of EC and explore the potential of ROS-targeted therapeutic strategies (Tables 1–3).

**Table 1:** Factors affecting endometrial cancer progression.

Factors promoting EC progression	Factors inhibiting EC progression
Amentoflavone Ellipticine Excessive ROS Hinokitiol Hydrogen Maackiain Methoxyeugenol Oleanolic acid 3-acetate Progesterone Simvastatin *α*-Terthienylmethanol	CaBP-28 k MAPK pathway (prior to inhibition) Estrogen-induced mitochondrial ROS NLRP3 ↓ PKD2, PRDX2, AQP1, TXNIP ROS SFA + insulin S1P ↑

AQP1, Aquaporin 1; CaBP-28k, Calcium-binding protein 28 kDa; MAPK, Mitogen-Activated Protein Kinase; NLRP3, NOD-like receptor family pyrin domain-containing 3; PKE2, Polycystic Kidney Disease 2; PRDX2, Peroxiredoxin 2; ROS, Reactive Oxygen Species; S1P, Shpingosine-1-phosphate; SFA, Saturated Fatty Acids; TXNIP, Thioredoxin Interacting Protein.

**Table 2:** Impact of ROS-related factors on the progression of EC and underlying mechanisms.

Direction of effect on EC progression	ROS-related Factor	Effect on endometrial cancer progression and underlying mechanisms
Promoting EC	↑ ROS	DNA damage and mutation EMT/angiogenesis
	Estrogen-induced mitochondrial ROS	Ca^2+^ influx → lysosome activity ↑
	PKD2/PRDX2/AQP1	Oxidative stress pathway activation
	S1P ↑	Proliferation/survival ↑
	NLRP3 ↓	Immune evasion (M2 macrophage polarization)
	CaBP-28 k ↑	Apoptosis suppression/ROS buffering
	Insulin + SFA	ROS ↑ → ER stress/mTOR pathway ↑
	RAS/MAPK pathway	ROS ↓ → Tumor promotion
Suppressing EC	Excessive ROS	Ferroptosis/apoptosis /pyroptosis
	Methoxyeugenol	ROS ↑ → p53/p21 ↑ → proliferation ↓
	Amentoflavone	ROS → AMPK/mTOR → ferroptosis ↑
	Maackiain	ROS → ER stress → apoptosis/autophagy
	Hydrogen	ROS → NLRP3/caspase-1 → pyroptosis
	Hinokitiol	ROS → Apoptosis/cell cycle arrest
	Progesterone	ROS ↓ → p53 ↑ → BCL-2 ↓
	Simvastatin	RAS/MAPK ↓ → ROS ↑ → ferroptosis ↑
	Ellipticine	ROS → MAPK → apoptosis ↑
	Oleanolic acid 3-acetate	ROS-independent mitochondrial apoptosis
	α-Terthienylmethanol	ROS ↑ (NADPH oxidase) → apoptosis

AMPK, AMP-activated protein kinase; AQP1, Aquaporin 1; BCL-2, B-cell lymphoma 2; CaBP-28k, Calcium-binding protein 28 kDa (Calbindin-D28k); EMT, Epithelial–mesenchymal transition; ER, Endoplasmic Reticulum; MAPK, Mitogen-Activated Protein Kinase; NLRP3, NOD-like receptor family pyrin domain-containing 3; PKD2, Polycystic Kidney Disease 2; PRDX2, Peroxiredoxin 2; ROS, Reactive Oxygen Species; S1P, Sphingosine-1-phosphate; SFA, Saturated Fatty Acids.

**Table 3:** Studies on reactive oxygen species and free radicals in endometrial cancer.

Author/Year/Reference	Study design	Species and/or sample	Detection method	Target substance(s) associated with ROS	Results/Conclusions
Blachnio-Zabielska A. U. et al. (2024)(25)	Comparative study	Patients (*N* = 37) who had undergone surgery for diagnosed type I endometrial cancer, FIGO stage 1, grade 2, and control group (*N* = 17), matched by BMI and age.	Sample collection, laboratory assessments, ultra-high-performance liquid chromatography coupled with tandem mass spectrometry, colorimetry, lipid peroxidation assay kit	MDA, sphingolipid	TOS in EC tissues was significantly higher than in the endometrium of healthy women (*p* < 0.0001), whereas TAC was significantly lower in EC compared to endometria in the control group (*p* < 0.01). The OSI was significantly higher in cancerous tissues compared with healthy endometrial tissues (*p* < 0.0001). MDA levels in EC were significantly higher (*p* < 0.0001) than in healthy endometrial tissue. A Pearson correlation analysis showed significant correlations between oxidative status and lipid concentration. /Endometrial cancer is characterized by profound changes in sphingolipid metabolism that contribute to oxidative dysregulation and tumor progression.
Costa B. P et al. (2021)(26)	*In vitro*	Ishikawa EC cell line	MTT assay, LDH-release assay, flow cytometry, RT-qPCR, antioxidant activity assay, DCFH-DA assay	ROS	Methoxyeugenol significantly inhibited the proliferation and viability of Ishikawa EC cells without inducing cytotoxicity. It also increased intracellular ROS levels and disrupted mitochondrial function, leading to upregulation of p53 and p21 and downregulation of CDK4/6, thereby arresting the cell cycle. The antiproliferative effect persisted even after treatment withdrawal, demonstrating long-term suppression of cell growth. /Methoxyeugenol exerts a potent anticancer effect against EC by modulating the p53/p21 pathway, increasing ROS levels, and inducing mitochondrial instability. This leads to cell cycle arrest and senescence, rather than apoptosis. These findings highlight methoxyeugenol as a promising candidate for EC therapy.
Mieszczanski P. et al. (2022)(27)	Comparative study	Patients (*N* = 45) diagnosed with EEC (endometrioid EC) and control patients (*N* = 45) without cancer	Oligonucleotide microarrays, RT-qPCR, ELISA, miRNA microarrays	ROS	PRDX2 and PKD2 protein were found to be overexpressed in EC, consistent with analyses at the mRNA level. Similar results were found for KLF2 expression, which was downregulated. Significantly altered expression of SOD3 (reduced) and AQP1 were found in G2 and G3 cancer. The reduced expression of SOD3 in G1 and G3 cancer may be attributable to the increased activity of miR-328 and miR-363, respectively. Reduced expression of KLF2 was associated with decreased miR-195-3p levels and overexpression of miR-363. /Oxidative stress is observed during the course of EC, and its regulation may be influenced by miRNAs.
Shen B. et al. (2022) (28)	Retrospective study	Patients (*N* = 502) diagnosed with EC after surgery, Ishikawa and AN3CA cells	Serum calcium analysis, proteomics, transcriptomics, cell culture, flow cytometry, cathepsin activity fluorometric assay, lyso-tracker red staining, acridine orange staining, Western blotting	Calcium, ROS	The degree of calcium influx depends on differences in extracellular calcium concentration at a constant estrogen stimulus. ROS production, confirmed by confocal microscopy and flow cytometry using the probes, DCFH-DA and Mito-SOX, was markedly reduced upon removal of extracellular calcium, underscoring the dependence of ROS generation on calcium influx. Proteomics and bioinformatics analyses revealed that calcium influx might be involved in the regulation of autophagy and mitochondrial-related pathways. /These findings emphasize the pivotal role of calcium influx and ROS in EC progression, highlighting potential targets for therapeutic intervention.
Nguyen H. et al. (2011) (29)	In vitro	EC cell lines ECC-1 and Ishikawa	MTS proliferation assay, Western blotting, fluorescence, immunoblotting	ROS, GPx1, SOD-1, catalase	ROS production was high in EC cells compared with their respective controls, and was markedly decreased by exposure to P4 for 6 h. Expression of nitrotyrosine and protein carbonyl was increased in EC cells compared with normal cells, but was decreased by treatment with P4 for 24 h. GPx1, SOD-1, catalase, and TRx1 expression were also higher in EC cells compared with control cells. Treatment of EC cells with P4 for 24 h suppressed all antioxidants studied. /P4 has antioxidant effects, alleviating ROS stress and causing apoptosis by inhibiting ROS production and decreasing protein carbonyl and lipid peroxidation metabolism.
Lampe R. et al. (2007) (30)	Comparative study	Blood collected from peripheral veins of female EC patients (N = 30) and healthy controls (N = 31)	Spectrophotometry	Superoxide anion	Superoxide anion production by granulocytes in the early stage of EC was lower (1.112 nM/min/10^5^ cells) than in controls. There was no significant difference in superoxide production between patients with different depths of myometrial infiltration. After treatment, superoxide anion by granulocytes from clinically tumor-free patients substantially increased (1.357 nM/min/10^5^ cells), and subsequently returned to a level lower than that in controls. /Damage to the non-specific immune response is instrumental in the development and clinical manifestation of EC and is already advanced at the earliest stage of EC.
Zhu X. et al. (2023)(31)	Animal study, *in vitro*	Mice with NLRP3-depleted macrophages and control mice transplanted with a human EMC cell line	ELISA, flow cytometry, ROS assay	ROS, NLRP3	Knocking out NLRP3 in macrophages shifted their polarization to a proinflammatory M2-like phenotype and significantly reduced ROS production. NLRP3 depletion in M2-polarized macrophages increased the growth, invasion, and metastasis of co-cultured EMC cells. NLRP3 depletion in M1-polarized macrophages reduced phagocytic potential, resulting in weakened immune defense against EMC. /These results underscore the critical role of NLRP3 in regulating macrophage polarization, oxidative stress, and immune defense mechanisms against EMC.
Chen, H. Y. et al. (2021) (32)	In vitro	Ishikawa, HEC-1A, and KLE EC cell lines	MTT assay, flow cytometry, cell viability assay, Western blotting	ROS, ERK1/2 pathway	Hinokitiol exerted potent anti-proliferative effects on EC cell lines (Ishikawa, HEC-1A, and KLE) by inducing apoptosis and cell cycle arrest. Treatment with hinokitiol significantly inhibited cell viability in a concentration-dependent manner, with IC_50_ values varying across cell lines. Hinokitiol also triggered apoptosis through caspase-3 activation, PARP cleavage and increased Bax/Bcl-2 ratio, suggesting involvement of the intrinsic (mitochondrial) apoptotic pathway. The compound also induced autophagy in Ishikawa and HEC-1A cells, as evidenced by LC3B-II accumulation and decreased p62 expression, although no significant autophagic response was observed in KLE cells. Furthermore, hinokitiol increased intracellular ROS levels, contributing to apoptosis and autophagy. /Hinokitiol demonstrates strong anticancer activity in EC cells by inducing cell cycle arrest, apoptosis, and autophagy, primarily via ROS generation and p53 activation. Its selective modulation of ERK signaling suggests differential regulatory mechanisms across EC subtypes. These findings support hinokitiol as a promising candidate for EC therapy, particularly in combination with ROS modulators or autophagy inhibitors.
Lee, J. S. et al. (2015)(33)	In vitro	HEC1A, Ishikawa EC cell lines	MTT assay, flow cytometry, annexin V-FITC/PI staining, Western blotting, ELISA	ROS	α-TM, a bioactive compound isolated from *Eclipta prostrata*, exhibited potent cytotoxicity against human EC cells (Hec1A and Ishikawa) with an IC₅₀ value of <1 μM. Mechanistically, *α*-TM significantly increased intracellular ROS levels while reducing GSH content, indicating oxidative stress. NAC, an ROS scavenger, effectively blocked α-TM–induced apoptosis, confirming that ROS generation plays a crucial role in its cytotoxicity. Further investigation revealed that NOX is a major source of ROS in α-TM–treated cells, as NOX inhibitors (DPI, apocynin) and siRNA targeting of p47phox significantly reduced ROS accumulation and apoptosis. However, inhibitors of MAPK pathways (ERK, JNK, and p38) had no significant effect, ruling out MAPK involvement. /α-TM exerts potent anticancer effects in EC cells by inducing apoptosis through ROS generation, primarily via NOX activation. Its pro-apoptotic effects are caspase-dependent and involve mitochondrial dysfunction, with cytochrome c release and amplification of oxidative stress. These findings suggest that α-TM is a promising natural compound for EC therapy, particularly in approaches targeting oxidative-stress–induced apoptosis.
Kim, J. Y. et al. (2011)(34)	In vitro	RL95-2 cell line	Cell viability assay, cell cycle analysis, Hoechst 33258 staining, annexin V cell death assay, immunocytochemistry, caspase-3 activity assay, MTT assay, DCF-DA assay	ROS, ERK, MAPK	Ellipticine exhibited strong pro-apoptotic effects in RL95-2 human EC cells by inducing cell cycle arrest at the G2/M phase, activating caspase-dependent and caspase-independent apoptotic pathways, and increasing ROS production. The ROS scavengers NAC and BHA significantly reduced ROS accumulation and inhibited caspase activation, ERK phosphorylation and AIF release, confirming that ROS play a critical role in ellipticine-induced apoptosis. Furthermore, inhibition of ERK with U0126 suppressed apoptosis and AIF release, indicating ERK’s involvement in the caspase-independent pathway. /Ellipticine effectively induces apoptosis in human EC cells via ROS generation, MAPK activation, and mitochondrial dysfunction. It activates both caspase-dependent and caspase-independent apoptosis, with ERK playing a key role in AIF release. These findings suggest that ellipticine is a promising therapeutic candidate for EC treatment, particularly in strategies targeting oxidative stress and apoptosis pathways.
Costa B. P. et al. (2021)(35)	In vitro	Ishikawa human endometrial adenocarcinoma cell line	Cell proliferation and cell viability assay, LDH-release assay, nuclear morphometric analysis, cell death assay, acidic vesicular organelle quantification, DPPH assay, DCFH-DA assay, clonogenic cell survival assay	F1,6BP, ROS	Treatment of Ishikawa cells with F1,6BP decreased cell number and cell viability in a concentration-dependent manner, without inducing cytotoxicity as measyred by membrane integrity assays (*p* < 0.0001). ROS levels in Ishikawa cells significantly increased in response to treatment with F1,6BP (p < 0.01). There was a strong positive correlation between ROS production and mitochondrial membrane potential. /F1,6BP acts as an anticancer agent through generation of mitochondrial instability, ROS production, loss of mitochondrial function, and promoting p53-dependent cell death.
Jo H. et al. (2020) (36)	In vitro	SKOV3, HEC-1A cells	Flow cytometry, immunoblotting, DCFH-DA assay	ROS	Oleanolic acid 3-acetate exhibited strong cytotoxic effects on ovarian (SKOV3) and endometrial (HEC-1A) cancer cells, reducing cell viability in a concentration-dependent manner and significantly inhibiting SKOV3 tumor growth in xenograft mice. Notably, oleanolic acid 3-acetate decreased ROS levels, indicating apoptosis occurs via a ROS-independent, but mitochondria-mediated, pathway. /Oleanolic acid 3-acetate demonstrates potential as an anticancer agent against ovarian and endometrial cancer by inducing apoptosis through a unique mitochondrial, but ROS-independent, mechanism. These findings highlight the therapeutic value of this compound as a novel supplement for cancer treatment.
Jung E. M. et al. (2011)(37)	In vitro	Ishikawa human endometrial adenocarcinoma cells	Cell culture, cell viability assay, TUNEL staining, Western blotting, RT-PCR	H_2_O_2_	Treatment of Ishikawa EC cells with 1 mM H_2_O_2_ for 1 h induced an increase in Bax and p53 expression, without affecting expression of Bcl-2. Overexpression of CaBP-28 k inhibited cell death and caused a decrease in Bax, p53 and caspase 3 expression during H_2_O_2_-induced apoptosis, suggesting that CaBP-28 k blocks up-regulation of apoptosis-related genes. siRNA-mediated knockdown of CaBP-28 k enhanced H_2_O_2_-induced cell death and increased Bax, p53 and caspase 3 expression, providing additional evidence that induction of CaBP-28 k gene expression might be associated with survival signaling during H_2_O_2_-induced cell death. /These results provide evidence that CaBP-28 k protects against apoptosis by regulating the pro-apoptotic genes, Bax and caspase-3, in H_2_O_2_-treated human endometrial adenocarcinoma cells.
Zhou J. et al. (2022) (38)	Retrospective study	Blood glucose and serum lipids of patients (N = 295) with newly diagnosed EC	TGGA analysis, cell culture, CCK-8 cell proliferation assay, confocal microscopy, Western blotting, comet assay, human phospho-kinase antibody array, RT-PCR	ROS	Insulin, a key regulator of glucose metabolism disorders, promoted DNA damage and disrupted ROS and Ca^2+^ homeostasis in a panel of established EC cell lines. Mitochondrial Ca^2+^ and ROS in EC cells increased within 10 min of insulin stimulation. Inhibition of ROS with NAC decreased insulin-induced DNA damage. Insulin also synergized with SFA to activate the mTOR/70 kDa ribosomal protein S6 kinase pathway and ER stress, resulting in Ca^2+^ release from the ER and activation of the UPR. /ER stress induced by combined treatment with insulin and SFA induced excessive Ca^2+^ entry in mitochondria in EC cells. Mitochondrial Ca^2+^ overload results in mitochondrial ROS overproduction.
Xing Y. et al. (2025) (39)	In vivo	KLE and Ishikawa human EC cell lines	CCK-8 cell viability assay, EdU detection, colony-formation assay, cell-cycle assay, FITC/PI staining, MMP detection, autophagy flux analysis, RNA-sequencing analysis, Western blot analysis, hematoxylin & eosin staining	ROS	Maackiain effectively inhibited the growth of EC cells in vitro and in vivo by inducing apoptosis and autophagy. Mechanistically, maackiain increased intracellular ROS levels, which triggered ER stress, leading to apoptosis and autophagy. The ROS scavenger, NAC, and ER stress inhibitor, 4-PBA, suppressed both apoptosis and autophagy, confirming that maackiain induces cell death through ROS-mediated ER stress. Additionally, autophagy played a cytoprotective role, as blocking it with chloroquine enhanced apoptosis, whereas activating it with rapamycin reduced cell death. /Maackiain plays a dual role in EC by inducing apoptosis and cytoprotective autophagy through ROS-mediated ER stress. These findings suggest that combining maackiain with autophagy inhibitors may enhance its anticancer efficacy, making it a promising therapeutic candidate for EC treatment.
Zhou D. et al. (2022) (40)	In vitro	EC cells (Ishikawa)	MTT assay, colony-formation assay, Transwell assay, ROS assay, biochemical kit-based assay, Western blotting	ROS, RAS/MAPK assays	Simvastatin exhibited potent anti-proliferative, anti-invasive, and ferroptosis-inducing effects in Ishikawa human EC cells by inhibiting the RAS/MAPK signaling pathway. Mechanistically, simvastatin increased intracellular ROS and MDA levels while reducing GSH levels, suggesting enhanced oxidative stress. It also induced ferroptosis, as evidenced by increased Fe^2+^ levels and upregulation of TRF1, a ferroptosis-promoting marker. Conversely, SLC7A11 and FPN, negative regulators of ferroptosis, were downregulated. /Simvastatin effectively suppresses EC progression by inhibiting the RAS/MAPK pathway, inducing oxidative stress, and promoting ferroptosis. The drug’s ability to reduce proliferation, colony formation, and invasion while increasing ROS and ferroptosis-related markers highlights its potential as a targeted therapy for EC. These findings suggest that simvastatin could be repurposed as an anticancer agent, particularly in combination with ferroptosis-inducing therapies.
Sun Q. et al. (2022) (41)	In vitro	KLE EC cell line	MTT assay, colony-formation assay, TUNEL assay, Western blotting, TBARS assay, DCFH-DA assay	ROS, AMPT/mTOR	Amentoflavone exerted potent anti-proliferative and pro-apoptotic effects in KLE human EC cells by inducing ferroptosis and regulating the ROS/AMPK/mTOR signaling pathway. Ferroptosis-related proteins were significantly modulated, with SLC7A11, GPX4, and FTH1 downregulated and ACSL4 upregulated, indicating enhanced ferroptosis. Mechanistically, amentoflavone activated AMPK while suppressing mTOR and promoting apoptosis and ferroptosis. This effect was dependent on ROS accumulation, as treatment with NAC, a ROS inhibitor, reversed amentoflavone-induced changes in AMPK/mTOR activity, cell proliferation, apoptosis, and ferroptosis markers, confirming that ROS/AMPK/mTOR signaling plays a central role in amentoflavone-induced cytotoxicity. /Amentoflavone exerts strong anticancer activity in EC by suppressing cell proliferation and promoting apoptosis and ferroptosis through modulation of ROS/AMPK/mTOR signaling. ROS accumulation is a key driver of amentoflavone-induced effects, as NAC treatment reversed its cytotoxicity.
Yang Y. et al. (2020) (42)	In vitro, in vivo	Ishikawa, AN3CA, and HEC1A EC cells lines, 4-week-old female specific pathogen-free grade BALB/c-nude mice	Immunohistochemical staining, Western blotting, immunoblotting, MTT assay, DCFH-DA assay, mitoSOX red assay, TUNEL assay, PI staining, ELISA, RT-qPCR	H_2_, ROS	H_2_ pretreatment upregulated ROS and the expression of pyroptosis-related genes, and increased the number of PI- and TUNEL- positive cells. It also promoted the release of LDH and IL-1β, effects reduced by GSDMD depletion. H_2_ supplementation in a xenograft mouse model exerted an anti-tumor effect, as evidenced by decreased tumor radiance, attenuating tumor volume and weight through the pyroptotic pathway. /H_2_ induces pyroptosis via a ROS-NLRP3-caspase-1 pathway. Consumption of H_2_-enriched water reduced the volume and weight of endometrial tumors in xenograft model mice.

ACSL4, acyl-CoA synthase long chain family member 4; AIF, apoptosis-inducing factor; AMPK, 5’ AMP-activated protein kinase; AQP1, aquaporin 1; Bax, Bcl-2-associated X protein; CaBP-28 k, calbindin-D28k; CDK4/6, cyclin-dependent kinase 4 and 6; DCFH-DA, 2′,7′-dichlorodihydrofluorescein diacetate; EC, endometrial cancer; ELISA, enzyme-linked immunosorbent assay; ERK, extracellular signal-regulated kinase; F1,6BP, fructose-1,6-bisphosphate; FPN, ferroportin; GPX4, glutathione peroxidase 4; GSH, glutathione; GSDMD, gasdermin D; LDH, lactate dehydrogenase; MAPK, mitogen-activated protein kinase; MDA, malondialdehyde; MEK, mitogen-activated protein kinase kinase; mTOR, mechanistic target of rapamycin; NAC, N-acetylcysteine; NOX, NADPH oxidase; OSI, oxidative stress index; PARP, poly(ADP ribose) polymerase; PCNA, proliferating cell nuclear antigen; P4, progesterone; PI, propidium iodide; PKD2, polycystin-2; PMNL, polymorphonuclear leukocytes; PRDX2, peroxiredoxin 2; p-ERK, phosphorylated ERK; p-MEK, phosphorylated MEK; qPCR, quantitative polymerase chain reaction; RAS, rat sarcoma virus oncogene; ROS, reactive oxygen species; RT-PCR, reverse transcription polymerase chain reaction; SFA, saturated fatty acid; SLC7A11, solute carrier family 7 member 11; SOD1, superoxide dismutase 1; SOD3, superoxide dismutase 3; TAC, total antioxidant capacity; TBARS, thiobarbituric acid reactive substance; TGF, transforming growth factor; TGGA, The Cancer Genome Atlas program; TOS, total oxidant status; TRF1, transferrin receptor 1; TRx1, thioredoxin 1; TUNEL, terminal deoxynucleotidyl transferase dUTP nick-end labeling.

### 3.1 The impact of ROS on EC progression

#### 3.1.1 ROS and oxidative stress

An analysis of endometrial tissues from patients with EC showed a consistent oxidative-stress signature alongside dysregulated sphingolipid metabolism: total oxidative status and malondialdehyde were increased, total antioxidant capacity was decreased, and multiple ceramide species (e.g., C14:0, C16:0, C18:1, C22:0, C24:0) were elevated relative to normal endometrium. The sphingosine-1-phosphate (S1P)/ceramide ratio—a surrogate for the balance between survival and death signaling—was reduced, and C22:0-ceramide positively correlated with oxidative-stress indices. Collectively, these data indicate that oxidative stress and sphingolipid remodeling co-occur in EC and may offer therapeutic entry points via ROS modulation and lipid pathway targeting (25).

#### 3.1.2 ROS, DNA damage, and genomic instability

In Ishikawa endometrial cancer (EC) cells, methoxyeugenol (MET, 60 μM) significantly reduced cell proliferation and viability while exerting a sustained antiproliferative effect without overt cytotoxicity. Mechanistically, MET increased intracellular ROS and mitochondrial membrane potential (ΔΨm), consistent with mitochondrial dysfunction, and shifted cell-cycle regulators toward arrest by upregulating p53 and p21 while downregulating CDK4/6. Morphologically, MET-treated cells displayed enlarged nuclei and an increased number of acidic vesicular organelles, indicative of a senescence-related response; however, *β*-galactosidase activity did not rise significantly, suggesting incomplete transition to a fully senescent state. Together, these findings support that MET suppresses EC cell growth through ROS generation, mitochondrial perturbation, and p53/p21-mediated cell-cycle inhibition, with minimal cytotoxicity (26).

Complementing these *in vitro* observations, an analysis of endometrial biopsy samples from patients with EC demonstrated broad oxidative-stress reprogramming at the gene and microRNA (miRNA) levels. PRDX2, PKD2 (polycystin-2), and AQP1 were overexpressed in tumor tissues, whereas SOD3 and KLF2 were downregulated. miRNA correlations suggested post-transcriptional regulation of these redox pathways: elevated PKD2 associated with reduced miR-195-3p, miR-20a, and miR-134; decreased SOD3 correlated with increased miR-328 and miR-363; and KLF2 downregulation tracked with suppression of miR-195-3p and overexpression of miR-363. PRDX2 and AQP1 upregulation appeared relatively miRNA-independent, implying alternative regulatory inputs (e.g., transcription-factor–driven control). Collectively, these data indicate that oxidative stress is a key pathogenic driver in EC and that specific miRNAs participate in modulating oxidative-stress–related gene expression, with implications for DNA damage responses and genomic instability (27).

#### 3.1.3 ROS and hormones: interaction with estrogen and progesterone

Endometrial cancer is a hormone-dependent malignancy in which estrogen and progesterone differentially modulate ROS metabolism. Estrogen has been reported to promote mitochondrial ROS production in EC cells, enhancing invasiveness and motility. In a study analyzing biopsy and blood samples from EC patients, albumin-corrected serum calcium levels were significantly higher than in controls (*p* < 0.05) and positively correlated with lymphovascular space invasion (LVSI), lymph node metastasis, myometrial invasion, and cervical involvement. *In vitro*, estradiol induced rapid Ca2 + influx from the extracellular milieu, disrupting intracellular calcium homeostasis and mitochondrial function, and significantly increasing ROS (*p* < 0.01). These findings support an estrogen–ROS–calcium axis whereby estrogen directly augments ROS generation, secondarily alters Ca2 + signaling and lysosomal activity, and promotes cancer cell survival and metastasis (28).

By contrast, progesterone (P4) appears to suppress ROS generation and induce apoptosis via antioxidant effects. In ovarian (OVCA 420, OVCA 429) and EC (ECC-1, Ishikawa) cell lines, treatment with P4 (10^−6^ M) reduced cell viability by 70–80% (*p* < 0.05); this effect was reversed by the progesterone receptor antagonist mifepristone. Baseline ROS, higher in cancer versus normal cells, declined significantly within 6 h of P4 exposure, and oxidative stress markers (protein carbonyls, nitrotyrosine, MDA) decreased by 24 h. P4 also reduced expression of antioxidant enzymes (GPx1, SOD-1, catalase, Trx1) and shifted apoptotic regulators toward cell death (p53 and BAX up; BCL-2 down; *p* < 0.05), indicating apoptosis induction concomitant with ROS attenuation. Collectively, these data suggest that P4 possesses antioxidant and anticancer properties relevant to endometrial and ovarian cancers (29).

#### 3.1.4 ROS and immune dysregulation

Granulocyte-derived oxidative activity appears blunted in EC. In a cohort study, superoxide anion production in patients’ granulocytes was significantly lower than in healthy controls (1.014 vs. 1.541 nM/min/10^5^ cells; *p* = 0.007). Among patients clinically tumor-free for >1 year, levels increased (1.357 nM/min/10^5^ cells; *p* = 0.05) yet remained below control values (*p* = 0.01), and were not associated with myometrial invasion depth. These results suggest that innate immune dysfunction—manifested as reduced granulocyte superoxide—may precede or facilitate tumor development rather than simply reflect disease burden, warranting studies on whether restoring this function has preventive or therapeutic value in EC (30).

At the level of tumor-associated macrophages, loss of the inflammasome component NLRP3 skews polarization toward an immunosuppressive M2 phenotype and diminishes ROS production. In EC tissues, NLRP3 expression was reduced; NLRP3 deficiency decreased macrophage ROS (*p* < 0.01), enhanced EC cell (HEC-1A) proliferation, invasion, and migration (all *p* < 0.05), and impaired phagocytic activity in M1 macrophages (*p* < 0.01). *In vivo*, adoptive transfer of NLRP3-deficient macrophages increased tumor growth and metastasis and reduced intratumoral CD3^+^CD8^+^ T-cell infiltration (all *p* < 0.05). Together, these findings indicate that NLRP3-dependent macrophage ROS supports effective antitumor immunity, and that therapeutic activation of NLRP3 or reprogramming of macrophage polarization may offer immunomodulatory strategies in EC (31).

### 3.2 Potential of ROS as a therapeutic target

#### 3.2.1 ROS and apoptosis

When ROS accumulate beyond a certain threshold, they can impair mitochondrial function, damage cellular membranes, activate inflammatory responses, and induce apoptosis, thereby exerting antitumor effects. Multiple studies have reported that increasing ROS levels can suppress the growth of EC cells and promote cell death.

##### 3.2.1.1 Hinokitiol

Using patient-derived EC cell lines (Ishikawa, HEC-1A, KLE), this study evaluated whether hinokitiol induces ROS-mediated apoptosis and p53-dependent cell-cycle arrest. Hinokitiol (5–50 μM) significantly reduced cell viability in a concentration-dependent manner (*p* < 0.001) and caused G0/G1 arrest. It increased the expression of the tumor suppressor p53 (*p* < 0.05) while decreasing the cell-cycle regulators CDK4 and cyclin D1 (both *p* < 0.05). Annexin V/PI staining showed significant increases in early and late apoptotic populations (*p* < 0.05). Western blotting further revealed elevated cleaved caspase-3 and cleaved PARP (poly (ADP-ribose) polymerase), along with an increased Bax/Bcl-2 ratio (all p < 0.05), consistent with activation of the mitochondrial apoptosis pathway. Hinokitiol also significantly increased intracellular ROS (*p* < 0.001), an effect associated with activation of ERK1/2 (extracellular signal-regulated kinase 1 and 2) signaling. In summary, hinokitiol promotes intracellular ROS accumulation, upregulates p53, activates ERK1/2, and induces apoptosis and G0/G1 cell-cycle arrest in EC cells, supporting its potential as a ROS-targeting anticancer agent (32).

##### 3.2.1.2 *α*-Terthienylmethanol

A study investigating α-terthienylmethanol (α-TM), a compound isolated from *Eclipta prostrata*, showed that α-TM induces apoptosis in EC cells by generating ROS via NADPH oxidase (NO😆). Treatment with α-TM produced a concentration-dependent decrease in viability in Hec1A and Ishikawa cells (IC50 < 1 μM; *p* < 0.001) and a significant increase in apoptosis (*p* < 0.001). Apoptosis was confirmed by annexin V/PI staining and by activation of caspase-3, −8, and −9 (*p* < 0.05), along with increased cytosolic cytochrome c release (*p* < 0.05). Intracellular ROS rose significantly after *α*-TM exposure (*p* < 0.001), whereas glutathione (GSH) levels declined (*p* < 0.01), indicating oxidative stress. Pretreatment with the ROS scavenger N-acetylcysteine (NAC) or catalase suppressed *α*-TM–induced apoptosis (*p* < 0.01), confirming ROS dependence. Moreover, NOX inhibitors (DPI, apocynin) reduced both ROS generation and apoptosis (*p* < 0.05), implicating NOX as a key upstream regulator. These findings indicate that α-TM promotes NOX-mediated ROS production and triggers caspase-dependent, mitochondrial apoptosis in EC cells, supporting its potential as a novel anticancer agent (33).

##### 3.2.1.3 Ellipticine

In EC RL95-2 cells, ellipticine was shown to induce ROS generation and activate MAPK signaling to promote apoptosis. Treatment with ellipticine (1–10 μM) significantly reduced cell viability in a concentration-dependent manner (*p* < 0.001), caused G2/M cell-cycle arrest, increased cyclin B1 (*p* < 0.05), and decreased cyclin D and p27 (both *p* < 0.05). Apoptosis was confirmed by activation of caspases-8, −9, −3, and −7 (*p* < 0.05) and PARP cleavage (*p* < 0.05), indicating a caspase-dependent component. Ellipticine also decreased mitochondrial membrane potential (ΔΨm; *p* < 0.05) and promoted cytosolic release of cytochrome c and AIF (*p* < 0.05), consistent with mitochondrial dysfunction associated with ROS accumulation. ERK and JNK phosphorylation increased significantly (*p* < 0.05); notably, ERK inhibition with U0126 reduced ellipticine-induced apoptosis and caspase-3 activation (*p* < 0.05), and ROS scavengers (NAC, BHA) suppressed ERK/JNK phosphorylation, indicating that ROS act upstream of MAPK activation. AIF release was inhibited by both ERK blockade and ROS scavenging (*p* < 0.05), suggesting a concurrent caspase-independent pathway. Collectively, these findings indicate that ellipticine induces G2/M arrest and apoptosis in EC cells via ROS accumulation and MAPK pathway activation, supporting its potential as an anticancer strategy (34).

##### 3.2.1.4 Fructose-1,6-bisphosphate

In Ishikawa EC cells, fructose-1,6-bisphosphate (F1,6BP; 300 μM) significantly suppressed cell proliferation and viability (both *p* < 0.05) without causing cytotoxicity or membrane damage. F1,6BP increased intracellular ROS (*p* < 0.01) and mitochondrial membrane potential (ΔΨm; *p* < 0.01), consistent with mitochondrial dysfunction. Apoptosis analysis showed a significant rise in late apoptotic cells (*p* < 0.01) with no evidence of necrosis (*p* > 0.05). Autophagy was also robustly induced (*p* < 0.001), as indicated by autophagosome formation. At the molecular level, p53 and BAX mRNA expression increased (both *p* < 0.05), confirming activation of a p53-dependent apoptotic pathway. In colony-formation assays, F1,6BP significantly reduced clonogenic potential (*p* < 0.05), indicating durable antiproliferative effects. Collectively, these findings suggest that F1, 6BP promotes apoptosis and autophagy in EC cells via ROS generation and mitochondrial dysfunction, supporting its potential as an anticancer candidate (35).

##### 3.2.1.5 Oleanolic acid 3-acetate

A study analyzing the anticancer effects and apoptosis-inducing mechanisms of oleanolic acid 3-acetate in EC (HEC-1A) cells found that this compound significantly decreased cell viability in a concentration-dependent manner compared to the control group (IC₅₀: 0.8 μM; *p* < 0.05) and activated the mitochondrial apoptosis pathway. It also significantly reduced cell viability in a concentration-dependent manner in ovarian cancer (SKOV3) cells (*p* < 0.05), as demonstrated by MTT assays. Both early and late apoptotic cell populations were significantly increased (*p* < 0.05), as demonstrated by annexin V/PI staining, and loss of ΔΨm was observed, indicating mitochondrial dysfunction. Western blot analysis confirmed mitochondrial cytochrome c release into the cytosol and nuclear translocation of AIF, demonstrating activation of both caspase-dependent and -independent apoptotic pathways. Immunoblotting further revealed activation of caspase-3/7/8, increased PARP cleavage, and reduced expression of Bcl-2, confirming that intrinsic mitochondria-mediated apoptosis was the main mechanism of action. Interestingly, unlike most anticancer agents that induce apoptosis through ROS generation, oleanolic acid 3-acetate significantly reduced ROS levels (*p* < 0.05), strongly suggesting a ROS-independent mechanism of cell death. In an *in vivo* xenograft model, SKOV3 tumor-bearing immunodeficient mice treated with 10, 20, or 40 mg/kg oleanolic acid 3-acetate for 3 weeks showed dose-dependent reductions in tumor size and weight (*p* < 0.05), with tumor inhibition rates of 32.5, 38.5, and 46.0%, respectively. No significant changes in body weight or signs of toxicity were observed, indicating good anticancer efficacy as well as safety. These findings indicate that oleanolic acid 3-acetate induces apoptosis by modulating mitochondrial function without elevating ROS, suggesting utility as an adjunct therapeutic for endometrial and ovarian cancers, potentially including contexts resistant to oxidative-stress–based therapies (36).

##### 3.2.1.6 CaBP-28k

The role of calbindin-D28k (CaBP-28k), a Ca^2+^-binding protein, in hydrogen peroxide (H₂O₂)-induced apoptosis was examined in a study using Ishikawa EC cells. Treatment with H₂O₂ (1 mM) significantly increased the expression of Bax, p53, and caspase-3 (*p* < 0.05), indicating activation of an apoptotic pathway associated with ROS accumulation. Notably, overexpression of CaBP-28 k significantly suppressed H₂O₂-induced upregulation of Bax, p53, and caspase-3 (*p* < 0.05), resulting in increased cell survival. In contrast, H₂O₂ treatment of cells in which CaBP-28 k was knocked down by small interfering RNA (siRNA) enhanced the expression of these apoptotic factors (*p* < 0.05) and led to decreased cell viability. These findings suggest that CaBP-28 k protects EC cells by modulating ROS-mediated apoptotic signaling and may represent a relevant component of ROS-targeted therapeutic strategies in EC (37).

#### 3.2.2 ROS and ferroptosis

Ferroptosis—a regulated, iron-dependent cell death driven by lipid peroxidation—can be triggered by ROS accumulation and has emerged as a therapeutic avenue in EC, particularly when antioxidant defenses are compromised. The potential of targeting ROS accumulation and ferroptosis in EC therapy was explored in a study that analyzed clinical data from EC patients and results obtained in immortalized, patient-derived Ishikawa EC cells. Insulin and saturated fatty acids (SFA) increased ROS in immortalized, patient-derived EC cells (Ishikawa), disrupted mitochondrial Ca^2+^ homeostasis, and activated the unfolded protein response (UPR), with upregulation of ER-stress markers such as CHOP/DDIT3 and GRP78/HSPA5. Pharmacologic attenuation of ER stress or mitochondrial Ca^2+^ uptake reduced ROS levels and suppressed ferroptosis and cell death, indicating that ROS-mediated ER stress and Ca^2+^ imbalance are critical upstream triggers of ferroptosis in this context (38).

Consistently, the isoflavonoid maackiain inhibited EC cell proliferation (Ishikawa, KLE) in a concentration-dependent manner while markedly elevating ROS. Rising ROS was accompanied by robust ER-stress activation (e.g., GRP78, phosphorylated PERK, ATF4, CHOP, phosphorylated IRE1α, and spliced XBP1), and both a ROS scavenger (NAC) and an ER-stress inhibitor (4-PBA) blunted maackiain-induced ferroptosis and cell death. Notably, autophagy functioned as a protective response: chloroquine enhanced, whereas rapamycin reduced, cell death. These data support a model in which ROS drives ER stress to promote ferroptosis, with concomitant autophagy buffering cytotoxicity; therapeutically, combining ferroptosis induction with autophagy inhibition may enhance efficacy. Overall, convergent evidence indicates that targeting ROS accumulation and ER-stress signaling can engage ferroptosis in EC, and that co-modulation of Ca^2+^ handling and autophagy may further optimize this strategy (39).

##### 3.2.2.1 Simvastatin

In Ishikawa endometrial cancer (EC) cells, simvastatin was evaluated for its effects on proliferation, invasion, and colony formation, as well as its ability to induce iron-dependent cell death (ferroptosis) via modulation of the RAS/MAPK pathway. Treatment with simvastatin (10–15 μM) significantly reduced cell proliferation, colony formation, and invasive capacity (all *p* < 0.01). Intracellular ROS, malondialdehyde (MDA), and Fe^2+^ levels increased (all *p* < 0.01), while glutathione (GSH) decreased (*p* < 0.01). Consistent with ferroptosis, SLC7A11 (cystine–glutamate antiporter) and ferroportin were downregulated (*p* < 0.01), and transferrin receptor 1 (TRF1) was upregulated (*p* < 0.01). Simvastatin also suppressed the RAS/MAPK pathway, lowering RAS expression and levels of phosphorylated MEK and ERK (all *p* < 0.01). Notably, co-administration of the RAS activator ML-098 partially reversed simvastatin’s anticancer effects. Together, these findings indicate that simvastatin inhibits EC cell proliferation and invasion through RAS/MAPK pathway suppression and promotes cell death by inducing ferroptosis, supporting its potential as a targeted therapeutic agent in EC (40).

##### 3.2.2.2 Amentoflavone

In KLE endometrial cancer (EC) cells, the flavonoid amentoflavone was evaluated for its ability to induce ferroptosis via the ROS/AMPK/mTOR pathway. Amentoflavone (50, 75, 100 μM) significantly decreased cell viability and proliferation and increased cell death in a concentration-dependent manner (all *p* < 0.001). TUNEL staining and immunoblotting showed increased Bax, cleaved caspase-3, and cleaved caspase-9, with concomitant reduction of Bcl-2 (all *p* < 0.001), indicating apoptosis. Markers of ferroptosis were also engaged: intracellular Fe^2+^ and lipid peroxidation (TBARS) were elevated (*p* < 0.001), while SLC7A11, GPX4, and FTH1 were downregulated and ACSL4 was upregulated (all *p* < 0.001). Mechanistically, amentoflavone increased ROS (*p* < 0.01), enhanced AMPK phosphorylation (*p* < 0.001), and reduced mTOR phosphorylation (*p* < 0.001). Notably, co-treatment with the ROS scavenger NAC suppressed amentoflavone-induced ferroptosis and cell death (*p* < 0.01) and reversed the AMPK/mTOR signaling changes, confirming ROS dependence. Overall, amentoflavone induces both ferroptosis and apoptosis by activating the ROS/AMPK/mTOR axis and shows potential as a therapeutic candidate for EC (41).

#### 3.2.3 ROS and pyroptosis

Beyond apoptosis and ferroptosis, pyroptosis—an inflammatory form of programmed cell death—has emerged as a potential therapeutic strategy in EC. In a study that investigated whether hydrogen (H₂) induces ROS accumulation and subsequently triggers pyroptosis in EC cells, EC cell lines (Ishikawa and HEC-1A) were treated with H₂, and the levels of ROS as well as expression of the pyroptosis-related proteins, NLRP3, caspase-1, and GSDMD (gasdermin D), were measured before and after treatment. The essential role of this pathway was further evaluated by knocking down GSDMD expression using shRNA, and the effects of H₂ on tumor growth were assessed in an *in vivo* mouse model. The study showed that treatment with H₂ significantly increased intracellular ROS levels (*p* < 0.05) and enhanced the expression of NLRP3, caspase-1, and GSDMD (p < 0.05), promoting pyroptotic cell death. Propidium iodide (PI) and TUNEL staining confirmed increased pyroptosis, whereas elevated lactate dehydrogenase (LDH) and interleukin (IL)-1β release indicated activation of inflammatory cell death. Notably, shRNA-mediated knockdown of GSDMD expression significantly reduced the release of LDH and IL-1β (*p* < 0.05) and inhibited pyroptosis, even with H₂ treatment. In the mouse model, H₂ exposure significantly inhibited endometrial tumor growth compared to controls (*p* < 0.01) and increased expression of NLRP3, caspase-1, and GSDMD, confirming the promotion of pyroptosis. These findings suggest that H₂ promotes ROS accumulation and triggers pyroptosis, offering a potential therapeutic strategy for EC. Collectively, these findings indicate that H₂ promotes ROS accumulation and triggers NLRP3–caspase-1–GSDMD–dependent pyroptosis in EC, suggesting a therapeutic avenue that could be combined with ferroptosis- or apoptosis-based strategies to enhance antitumor efficacy (42).

### 3.3 Limitations

This review has several limitations. First, most of the evidence synthesized derives from laboratory studies; additional preclinical and clinical investigations are required to establish translational relevance in real-world settings. Given the dual and context-dependent roles of ROS in both cancer progression and cell death, it remains essential to determine how ROS modulation operates therapeutically in defined patient subgroups rather than assuming uniform benefit from increasing or decreasing ROS levels. Second, our analysis focused on selected EC models; therefore, further work is needed to confirm generalizability across the histologic and molecular heterogeneity of EC. Subtype-specific ROS dependencies likely exist, and mechanisms that induce apoptosis or ferroptosis in one context may be attenuated or redirected in another. Expanding research to encompass diverse EC subtypes and tumor–microenvironment interactions will be critical. Third, whether ROS-targeted strategies move beyond short-term efficacy to improve durable outcomes (resistance, recurrence) remains uncertain. Longitudinal studies with pharmacokinetic/pharmacodynamic readouts and normal-tissue safety assessments are needed in parallel to ensure that modulation of pleiotropic ROS pathways does not harm non-cancerous endometrium or other organs. Fourth, although some ROS-related mechanisms may be shared across gynecologic malignancies, this review was scoped to EC and did not conduct a systematic cross-cancer synthesis. Consequently, we cannot delineate which mechanisms are common versus lineage-specific across endometrial, ovarian, and cervical cancers. Notably, even within gynecologic models, ROS contributions can diverge: oleanolic acid 3-acetate elicits potent, mitochondria-mediated apoptosis while lowering ROS in ovarian (SKOV3) and endometrial (HEC-1A) systems, contrasting with EC agents such as ellipticine and hinokitiol that induce ROS-dependent apoptosis via MAPK/ERK or p53-linked signaling. Structured cross-cancer comparisons are needed to define shared versus lineage-restricted ROS dependencies and to inform biomarker-guided patient selection. Fifth, pathway pleiotropy and network cross-talk complicate target specificity. Modulating ROS can influence multiple intracellular signaling axes (e.g., AMPK/mTOR, RAS/MAPK, inflammasome pathways), raising the potential for off-target effects and context-dependent antagonism. Before clinical translation, strategies that enhance target precision and clarify mechanism—potentially by integrating ferroptosis or pyroptosis readouts with pathway-selective perturbations—should be prioritized.

## 4 Conclusion

This literature review reveals that ROS play pivotal roles in the pathophysiology of EC, functioning dually to both promote tumor progression and induce apoptotic cell death. ROS influence cancer cell growth and responsiveness to anticancer therapies by modulating the expression of specific genes. Notably, miRNAs appear to play an important regulatory role in this process, suggesting their potential involvement in ROS-mediated signaling pathways.

Furthermore, the interaction between ROS and ER stress, as well as the relationship between ROS and ferroptosis, highlight novel therapeutic targets in the treatment of EC. Given the close relationship between metabolic disorders and the development and progression of EC, therapeutic strategies that regulate oxidative stress while preserving normal cellular function are warranted. Such approaches may offer a means for overcoming the limitations of current therapies for EC. This study underscores the fact that oxidative stress acts as a key regulatory factor in the progression of EC and suggests that targeting ROS may be an effective therapeutic strategy. Future research should aim to elucidate the mechanisms of ROS regulation in greater detail and to develop personalized treatment approaches based on these findings. Ultimately, this could lead to novel therapies that enhance treatment efficacy while minimizing side effects in patients with EC.
